# Portable X-ray fluorescence of zinc and selenium with nail clippings–Mother and Infant Nutrition Investigation (MINI)

**DOI:** 10.1371/journal.pone.0310845

**Published:** 2024-10-23

**Authors:** David E. B. Fleming, Nelly Madani, Michaela G. Kaiser, Jong Sung Kim, Erin Keltie, Natashia Drage, Ying Jin, Jane Coad, Louise Brough

**Affiliations:** 1 Physics Department, Mount Allison University, Sackville, New Brunswick, Canada; 2 Department of Community Health and Epidemiology, Dalhousie University, Halifax, Nova Scotia, Canada; 3 Current address: Department of Occupational and Environmental Health, University of Iowa, Iowa City, Iowa, United States of America; 4 School of Health Sciences, Massey University, Palmerston North, New Zealand; 5 School of Food and Advanced Technology, Massey University, Palmerston North, New Zealand; Harvard School of Public Health, UNITED STATES OF AMERICA

## Abstract

Zinc and selenium are essential minerals for human nutrition. Reliable biomarkers of zinc status and selenium status in humans are therefore important. This work investigates a novel portable X-ray fluorescence (XRF) method with the ability to rapidly assess zinc and selenium in nail clippings. This approach used a mono-energetic X-ray beam to excite characteristic X-rays from the clippings. Nail clippings were obtained from the Mother and Infant Nutrition Investigation (MINI), a study designed to assess nutrition in a population of women and their breastfed children in New Zealand. Twenty mother-infant pairings were selected to provide nail clippings at two time points (visit 1 at 3 months postpartum; visit 2 at 6 months postpartum). Nail clippings from each mother-infant pairing were divided into three groupings of clippings prior to analysis: those obtained from a big toe of the mother, those from the other toes of the mother, and those from the toes and fingers of the infant. Clippings were prepared and mounted prior to XRF measurement, providing four distinct fragments from each clipping grouping. These fragments were assessed by XRF using a measurement time of either 300 s (visit 1) or 180 s (visit 2). XRF results were determined through both an automated system output and an analysis of the X-ray energy spectrum. Following this assessment of zinc and selenium with the non-destructive XRF method, clippings were measured for zinc and selenium concentration using a “gold standard” technique of inductively coupled plasma mass spectrometry (ICP-MS). Mean ICP-MS concentrations ranged from 122 μg/g to 127 μg/g for zinc, and from 0.646 μg/g to 0.659 μg/g for selenium. Precision, assessed by a relative standard deviation of measurement, was superior for ICP-MS relative to XRF. For both zinc and selenium, XRF results were compared with ICP-MS concentrations. Linear equations of best fit were determined for each comparison between XRF and ICP-MS results. Coefficients of determination (r^2^) were stronger for zinc (from 0.74 to 0.95) than selenium (from 0.53 to 0.70). A decrease in XRF measurement time from 300 s to 180 s did not appear to adversely affect the correlation between XRF and ICP-MS results. Using the mono-energetic portable XRF method, the correlation of XRF zinc results with ICP-MS zinc concentrations was improved over previous findings, and selenium measurement was reported for the first time. The method may prove useful for future applications to trace element analysis using nail clippings as a biomarker.

## Introduction

Zinc and selenium are two essential minerals for human nutrition. Deficient intake of either element can result in a variety of deleterious health effects. Zinc deficiency has been linked to adverse effects on growth and development [1] as well as the impairment of the gastrointestinal [2], immune [3], and central nervous [4] systems. Selenium deficiency has been associated with reduced capacity for brain function [5], increased risk of depression (and postpartum depression, in particular) [6], elevated risk of oxidative damage in the thyroid [7], and suboptimal levels of thyroid hormone (normally produced through interactions of selenium with iodine and iron) [8].

For these reasons, the search for reliable biomarkers of zinc status and selenium status in humans has become important. The current paper explores the use of a relatively new method of analysis for both zinc and selenium when using nail clippings as a biomarker. Nail clippings are simple to obtain, store, and transport [9]. They are also relatively free of external contamination [10] and can be cleaned [11] to help guard against this concern. Due to the growth rates of nail, nail clippings indicate exposure months in the past and over a longer time frame than other biomarkers [10]. For some elements, nail clippings are already well characterized and established as a useful biomarker [10, 12]. Here, we briefly summarize a series of review papers specifically examining biomarker candidates for zinc and selenium. For zinc status, plasma, urine, and hair concentrations were noted as reliable biomarkers in one review [13]. Another review recommended dietary intake, plasma zinc, and height-for-age in children as useful indicators [14]. Hair zinc, urine zinc, and neurobehavioral function were classified as potentially useful markers, while nail zinc concentration was noted as an emerging marker [14]. For selenium, the following biomarkers were identified as responding to changes in intake: plasma, erythrocyte, and whole-blood selenium; plasma selenoprotein P; and plasma, platelet, and whole-blood glutathione peroxidase [15]. Another review [16] noted that a series of potential biomarkers, including tissue, urine, and feces concentrations, could provide indirect inferences of selenium status, and that toenail selenium has been found to correlate well with plasma (or serum) and whole-blood selenium [17, 18].

While nail clippings are a possible biomarker for both zinc and selenium, the assessment of these trace elements in nail clippings through portable X-ray fluorescence (XRF) is a relatively new development [9]. As a method of analysis, portable XRF has some potential advantages over more conventional approaches such as inductively coupled plasma mass spectrometry (ICP-MS) or instrumental neutron activation analysis (INAA). These include its speed of measurement, simplicity in terms of both method and data analysis, low cost, non-destructive nature, and mobility [9]. However, the accuracy and precision of measurement with portable XRF is not expected to be as good as with conventional methods [9]. In this study, we will assess the capabilities of portable XRF for the assessment of both zinc and selenium in nail clippings by comparing XRF results with those from a “gold standard” technique, ICP-MS. The nail clipping samples to be used in this study present a good test of the portable XRF method as they span a range of masses and sizes.

The nail clippings for this study were obtained from a larger investigation examining the nutritional status of mothers and their infants in New Zealand. The Mother and Infant Nutrition Investigation (MINI) is a longitudinal cohort study designed to assess nutrition and related health outcomes in a population of women and infants from the Manawatu region of the North Island in New Zealand [19]. Breastfeeding women over 16 years of age with a healthy term infant were invited to participate in the study. Mother and infant study visits were made at 3, 6, and 12 months postpartum, with a variety of assessments performed [19]. These included dietary assessment and the collection of urine, blood, and breast milk samples from the mothers for the determination of intake/status for iodine, selenium, and iron. These three minerals are considered essential for optimal thyroid function in breastfeeding women. The assessment of iodine and selenium concentrations in urine were also performed for the infants. Toenail clippings were collected from the women at each of the three study visits, while fingernail and toenail clippings were collected from the infants. The overall MINI study had 87 mother-infant pairings participate [20, 21]. In recent years, iodine fortification and supplementation strategies have been introduced in New Zealand to combat the potential for iodine deficiency [20]. With respect to iodine status, it was found that the population of women enrolled in the study was in general iodine-deficient, and that the status of the infants was also suboptimal [20]. In terms of selenium intake, New Zealand has a history of low levels of selenium in the food supply [21] resulting from local soil conditions [22]. The MINI study found suboptimal intake of selenium in participating mothers and their infants, with the possibility of insufficient selenium status for some of the women [21]. It should be noted that the results outlined above from the MINI study pertaining to iodine and selenium were drawn from dietary, urine, blood, and breast milk assessments. To this stage, no analysis relating to nail clippings from this population has been published.

## Methods

### Nail clipping collection and preparation

Nail clippings were originally obtained from 87 mother-infant pairings participating in the MINI study. The MINI study protocol was approved by the Northern A Health and Disability Ethics Committee (New Zealand) and by the Mount Allison University Research Ethics Board (Canada). Informed written consent was obtained from each participant or their parent/guradian. The recruitment period began June 1, 2016 and ended December 31, 2017. Toenail clippings were obtained from the women, and both toenail and fingernail clippings from the infants. Following instructions provided by the investigators, nail clippings were self-collected by participants at home and brought to their next study visit [19]. Clippings were stored at room temperature while awaiting analysis. From the initial 87 mother-infant pairings participating in the full MINI study, 20 pairings were selected randomly for this comparative study to assess zinc and selenium in nail clippings using both portable XRF and ICP-MS. Clippings were washed and prepared for analysis through repeated cleanings with both acetone and water, using a technique similar to those described previously [11, 23]. Nail clippings from each mother-infant pairing were divided into three groupings of clippings prior to analysis: (1) those obtained from a big toe of the mother; (2) those from the other toes of the mother; and (3) those from the toes and fingers of the infant. With up to 20 mother-infant pairings available, this resulted in a maximum of 60 groupings of nail clippings for analysis from a given study visit.

### XRF method

**Fig 1:**
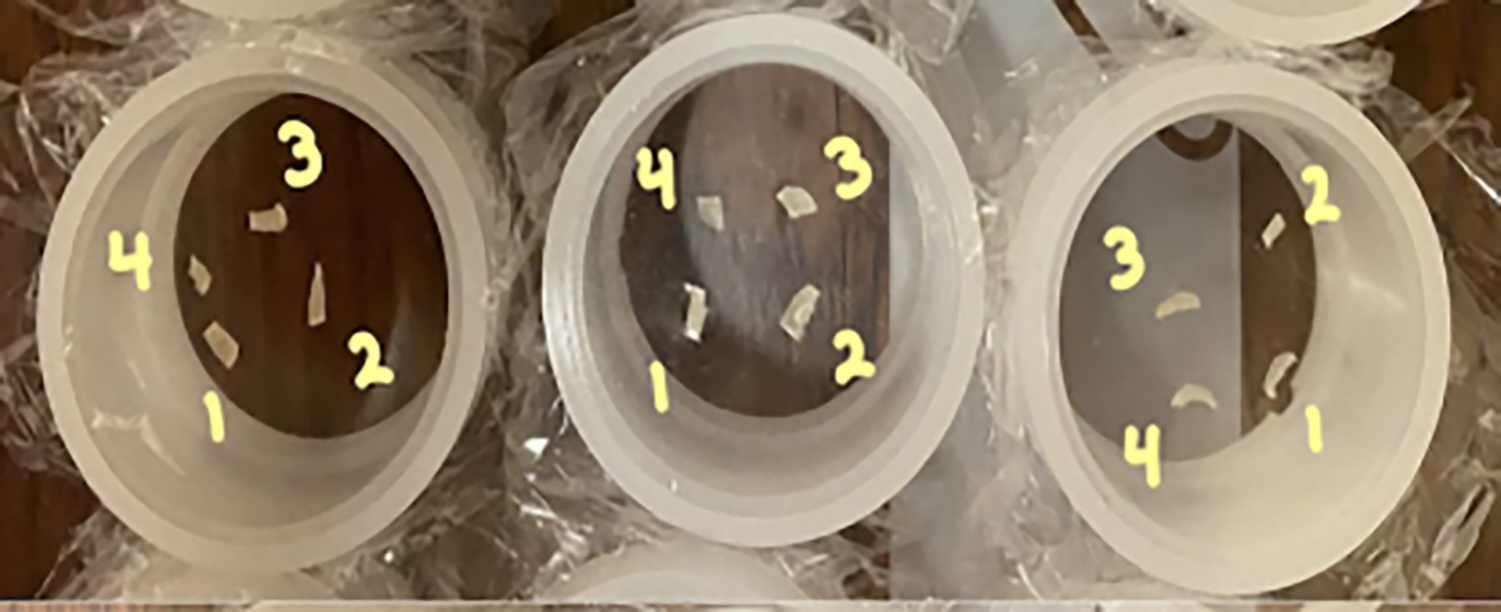
Sample cells containing nail clipping fragments prepared for XRF analysis. The three cells shown are from a single mother-infant pairing and contain, from left to right: fragments from a big toe of a mother, from other toes of a mother, and from toes and fingers of an infant. For each cell, the four fragments are labeled corresponding to the numerical order of their measurement.

A limited number of nail clippings from each of these groupings were selected for analysis by portable XRF. Individual clippings were chosen and trimmed with sterile nail clippers to obtain a smaller, relatively flat nail fragment. Typically, four separate fragments from four different nail clippings were used from each grouping of clippings. (In the rare instance where fewer than four clippings were provided, multiple fragments were obtained from one or more clippings.) The resulting collection of four nail fragments was then placed in an open-ended XRF sample cell between two sheets of 4 μm-thick prolene film (SCP Science; Baie-d’Urfé, QC, Canada). A typical collection of nail fragments in sample cells is shown in Fig 1. Each nail fragment was subsequently measured using an HD Mobile XRF system, operated in benchtop mode (X-ray Optical Systems; East Greenbush, NY, USA). The XRF system emitted X-rays from a molybdenum X-ray tube, operating with a ~200 μA tube current and a tube voltage ranging up to 50 kV. The HD Mobile system provided a ~17.5 keV mono-energetic X-ray beam through the use of X-ray optics. The beam size was 1 mm in diameter, small enough for the beam to entirely strike a single nail fragment within the sample cell. The radiation detector in the HD Mobile system was a silicon drift detector with an area of 25 mm^2^.

Individual measurements with the XRF system were made on each of the four nail fragments available in a sample cell. For the nail clippings obtained from study visit 1 (3 months postpartum), each XRF measurement lasted for 300 s (real time). Nail clippings obtained from study visit 2 (6 months postpartum) were measured for only 180 s (real time). Since there is no standardized practice for XRF measurement time with nail clippings, we were interested to test whether a shorter acquisition time would adversely affect the XRF results, as judged by a comparison with ICP-MS results. From study visit 1, a total of 20 mother-infant pairings were represented, producing a total of 60 sample cells for measurement. From study visit 2, only 18 mother-infant pairings provided complete collections of nail clippings, and one additional pairing provided infant clippings only. This resulted in a total of 55 sample cells for measurement from study visit 2. In each sample cell, the four nail fragments were measured individually, with both zinc and selenium being assessed. For reasons described below, the XRF results were analyzed in two different ways: (1) the automated factory-calibrated concentrations of zinc and selenium were obtained directly from the XRF system; and (2) the raw energy spectra, consisting of count detections as a function of energy, were obtained and exported for later analysis.

For the automated factory-calibrated XRF output, a concentration value and an uncertainty in concentration were provided from each of the four nail fragments for both zinc and selenium. These values were used to calculate a weighted mean average concentration result from the overall sample cell for zinc and for selenium. It should be noted that the factory calibration for the XRF system assumed a sample matrix (plastic) which was not equivalent to human nail. The concentration values output from the system were therefore not expected to be accurate, but still provided useful information for comparative purposes. In the case of the raw energy spectra, analysis of zinc and selenium signals proceeded through an external software package. The general approach used for XRF energy spectrum analysis has been described in detail in other nail clipping studies [24, 25] and will be summarized here. Energy spectra were analyzed using PyMca software, originally developed at the European Synchrotron Radiation Facility for the purpose of fitting and quantifying X-ray spectra [26, 27]. The following elements were found to contribute detection peaks in the energy range of interest and were therefore selected in the “fit configuration” window of PyMca: zinc, selenium, nickel, copper, arsenic, lead, and bromine. For each element (with the exception of lead), K-shell characteristic X-ray transitions were selected for the fitting. For lead, L-shell characteristic X-ray transitions were selected. After analysis with the software, each spectral fit was reviewed to confirm consistency with the observed data. The peak area and uncertainty in peak area were recorded from the zinc and selenium signals. A previously developed approach [28] was then used where each elemental signal was normalized against the total area under the entire detected energy spectrum. The results from this normalization approach are described as the total area ratio (TAR), a unitless quantity. TAR signals for both zinc and selenium, obtained from the four separate nail fragment measurements performed in each sample cell, were used to calculate a weighted mean XRF TAR result for zinc and a weighted mean XRF TAR result for selenium.

### ICP-MS method

For a given mother-infant pairing, nail clippings from each available grouping (big toe of the mother, other toes of the mother, toes and fingers of the infant) were then analyzed by ICP-MS to determine the concentrations of zinc and selenium. Since the combined masses of the four individual nail fragments from the XRF analysis noted above were relatively high for the big toe of the mother and for the other toes of the mother, the ICP-MS measurements used the same four fragments (and only these fragments) as the sample to analyze in each case. However, for the infant, the combined masses of the four individual nail fragments used with the XRF measurements were relatively low (sometimes below 5 mg). For this reason, all available infant nail clippings originally collected during the study visit were used as the sample for the ICP-MS measurements.

The method for ICP-MS analysis has been described previously [24, 25] and is briefly summarized here. Samples were initially weighed to determine a wet mass. After cleaning with acetone (99.5%, Acros Organics; Morris Plains, NJ, USA) and water using sonication, the samples were dried overnight in a convection oven (Heratherm 60 L gravity oven, Thermo Scientific; Waltham, MA, USA) at 105°C and then reweighed for their dry mass. Clipping samples were completely dissolved using a Discover SP-D microwave digestor (CEM Corporation; Matthews, NC, USA) in a mixture of 100 μL concentrated nitric acid (Fisher Scientific; Fair Lawn, NJ, USA), 400 μL water, and 500 μL hydrogen peroxide (Sigma Aldrich; St. Louis, MO, USA). After digestion, samples were diluted to 10 mL with water for a final nitric acid concentration of 1% (v/v) before ICP-MS analysis.

The total concentrations of zinc and selenium in the clipping samples were measured using an ICP-MS system (iCAP Q, Thermo Scientific; Waltham, MA, USA). The samples were introduced via an ESI SC-4 DX autosampler with a FAST valve (Elemental Scientific; Omaha, NE, USA), allowing for the online addition of a 50 μg/L scandium internal standard (AccuStandard; New Haven, CT, USA) in 1% (v/v) nitric acid. We used high-purity helium (>99.999%) as the collision gas and the kinetic energy discrimination (KED) mode of the instrument to eliminate polyatomic interference in the determination of zinc and selenium levels in the samples. A multi-element calibration standard (P/N IV-ICPMS-71A, Inorganic Ventures; Christiansburg, VA, USA) was diluted to concentrations ranging from 0.1 to 50 μg/L in 1% (v/v) nitric acid to establish a 7-point calibration curve. Quality control check standards (1 and 10 μg/L) were assessed after every 20 samples. The data from the ICP-MS analysis were collected and processed using Qtegra Intelligent Scientific Data Solution software (version 2.7, Thermo Scientific; Waltham, MA, USA).

### Statistical analysis

Calculations and statistical tests using data from the XRF and ICP-MS measurement results were performed using Microsoft Excel for Microsoft 365 (Microsoft Canada; Toronto, ON, Canada). In addition to the calculations described above, these included the provision of basic summary statistics such as the mean, sample standard deviation, and median of the ICP-MS results. Relative uncertainties of measurement were assessed for the XRF and ICP-MS techniques. Linear correlations between the XRF results and the gold standard ICP-MS concentration results were also examined, with coefficients of determination (r^2^) calculated in each case. When comparing zinc concentration results obtained from the XRF and ICP-MS methods, a Bland-Altman plot was also provided.

## Results

For the nail clipping samples obtained at visit 1 and visit 2 from the MINI study participants, zinc and selenium were measured by both portable XRF and ICP-MS. In the case of the ICP-MS measurements, zinc was detected in all 60 samples from visit 1 and all 55 samples from visit 2. Selenium was detected in 58 of the 60 samples from visit 1, and in all 55 samples from visit 2. Summary statistics from the ICP-MS concentration results are provided in Table 1 (zinc) and Table 2 (selenium). For this study, the individual sample ICP-MS concentrations will be considered the gold standard results against which the portable XRF results can be compared.

**Table 1:** Summary statistics from ICP-MS zinc concentration measurements of various groupings of nail clippings obtained from the MINI study.

	Visit 1	Visit 2
**N**	60	55
**Minimum**	69.3 μg/g	78.3 μg/g
**Maximum**	291 μg/g	342 μg/g
**Mean**	122 μg/g	127 μg/g
**Standard deviation**	36.5 μg/g	43.4 μg/g
**Median**	118 μg/g	118 μg/g

**Table 2:** Summary statistics from ICP-MS selenium concentration measurements of various groupings of nail clippings obtained from the MINI study.

	Visit 1	Visit 2
**N**	58	55
**Minimum**	0.211 μg/g	0.391 μg/g
**Maximum**	1.28 μg/g	0.996 μg/g
**Mean**	0.659 μg/g	0.646 μg/g
**Standard deviation**	0.262 μg/g	0.138 μg/g
**Median**	0.635 μg/g	0.643 μg/g

**Fig 2:**
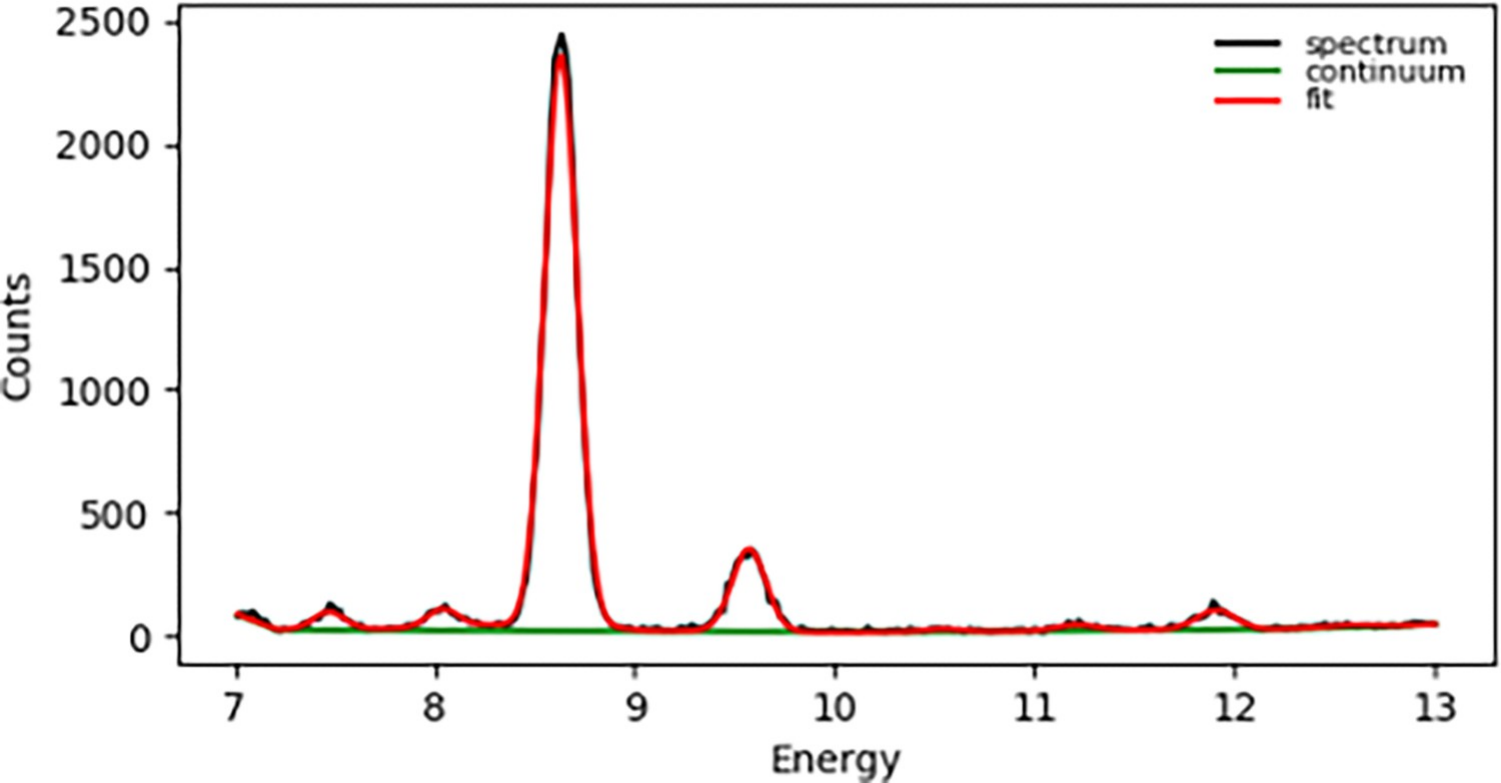
Energy spectrum from XRF measurement of a nail clipping. Counts are provided as a function of energy (keV). The red line indicates the PyMca fit for various elements, while the green line represents the background continuum. Observed data points are given in black and are mostly obscured by the red fit line.

A typical energy spectrum from portable XRF measurement of a single nail clipping is provided in Fig 2. The fitting of the spectrum data through PyMca analysis shows the clear detection of zinc characteristic X-rays at approximately 8.6 keV (Kα) and 9.6 keV (Kβ). The characteristic X-rays from selenium are much smaller in quantity, but a detection peak at approximately 11.2 keV (Kα) is still evident. As demonstrated by this spectrum, the XRF measurement system benefits from a very small and flat background continuum, aiding in the detection of relatively small signal peaks.

Uncertainty in measurement from the various methods used with the ICP-MS and XRF techniques is important to consider. Table 3 (zinc) and Table 4 (selenium) provide an overview of results for the relative uncertainties of measurement from the techniques used in this study. For each technique, a measurement uncertainty from an individual grouping of clippings was first determined from the sample standard deviation of the available measurements. The ICP-MS technique, for example, involved three separate injection runs for each sample, while both XRF analysis techniques involved four separate nail fragment measurements. The relative standard deviation from a grouping of clippings was then calculated by dividing the standard deviation by the actual measured value in each case. Typical sizes of the relative standard deviation from the various measurement techniques are summarized in Tables 3 and 4.

**Table 3:** Relative standard deviations for the measurement of zinc in nail clippings when determining concentration using ICP-MS, concentration using the automated XRF system output, and the total area ratio (TAR) from energy spectrum analysis using XRF. The table provides the mean, standard deviation (SD), standard deviation of the mean (SDOM), and median from both visit 1 and visit 2 measurements.

	ICP-MS	XRF system output	XRF TAR
**Visit 1 mean**	3.7%	11.7%	8.7%
**Visit 1 SD / SDOM**	2.4% / 0.3%	7.8% / 1.0%	5.5% / 0.7%
**Visit 1 median**	3.1%	9.8%	7.5%
**Visit 2 mean**	1.8%	14.6%	9.2%
**Visit 2 SD / SDOM**	1.3% / 0.2%	10.9% / 1.5%	4.4% / 0.6%
**Visit 2 median**	1.3%	10.3%	8.9%

**Table 4:** Relative standard deviations for the measurement of selenium in nail clippings when determining concentration using ICP-MS, concentration using the automated XRF system output, and the total area ratio (TAR) from energy spectrum analysis using XRF. The table provides the mean, standard deviation (SD), standard deviation of the mean (SDOM), and median from both visit 1 and visit 2 measurements.

	ICP-MS	XRF system output	XRF TAR
**Visit 1 mean**	5.5%	18.3%	14.3%
**Visit 1 SD / SDOM**	5.1% / 0.7%	18.0% / 2.4%	6.9% / 0.9%
**Visit 1 median**	4.4%	12.4%	13.3%
**Visit 2 mean**	3.2%	21.4%	16.1%
**Visit 2 SD / SDOM**	2.6% / 0.3%	16.5% / 2.2%	10.3% / 1.4%
**Visit 2 median**	2.5%	15.8%	14.7%

**Fig 3:**
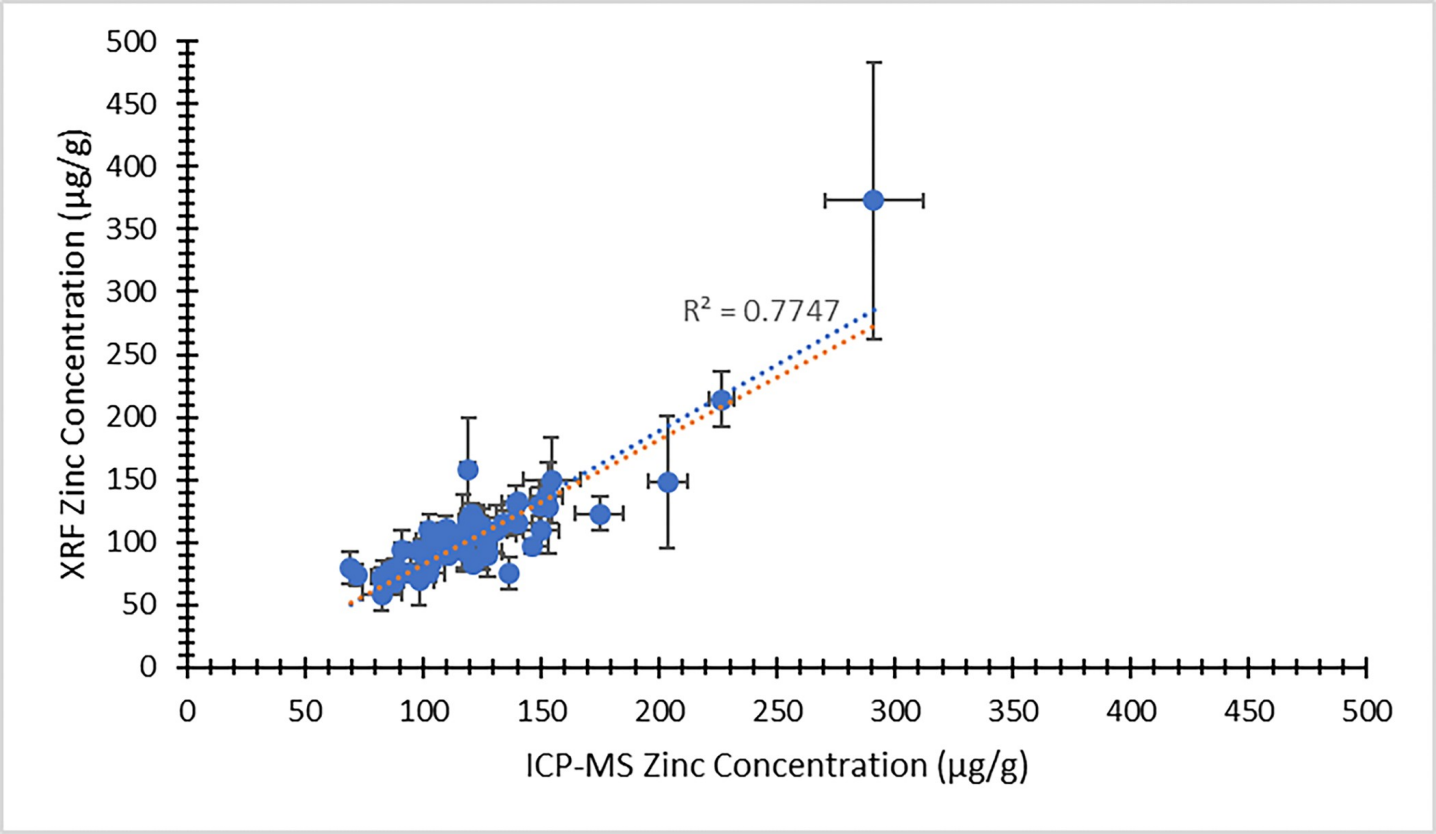
XRF system output zinc concentration (μg/g) as a function of ICP-MS zinc concentration (μg/g) for nail clippings from visit 1 of the MINI study. Uncertainty bars are provided for each data point. The unweighted best fit linear relationship between the variables is displayed as a blue dotted line and the weighted best fit linear relationship is displayed as an orange dotted line.

**Fig 4:**
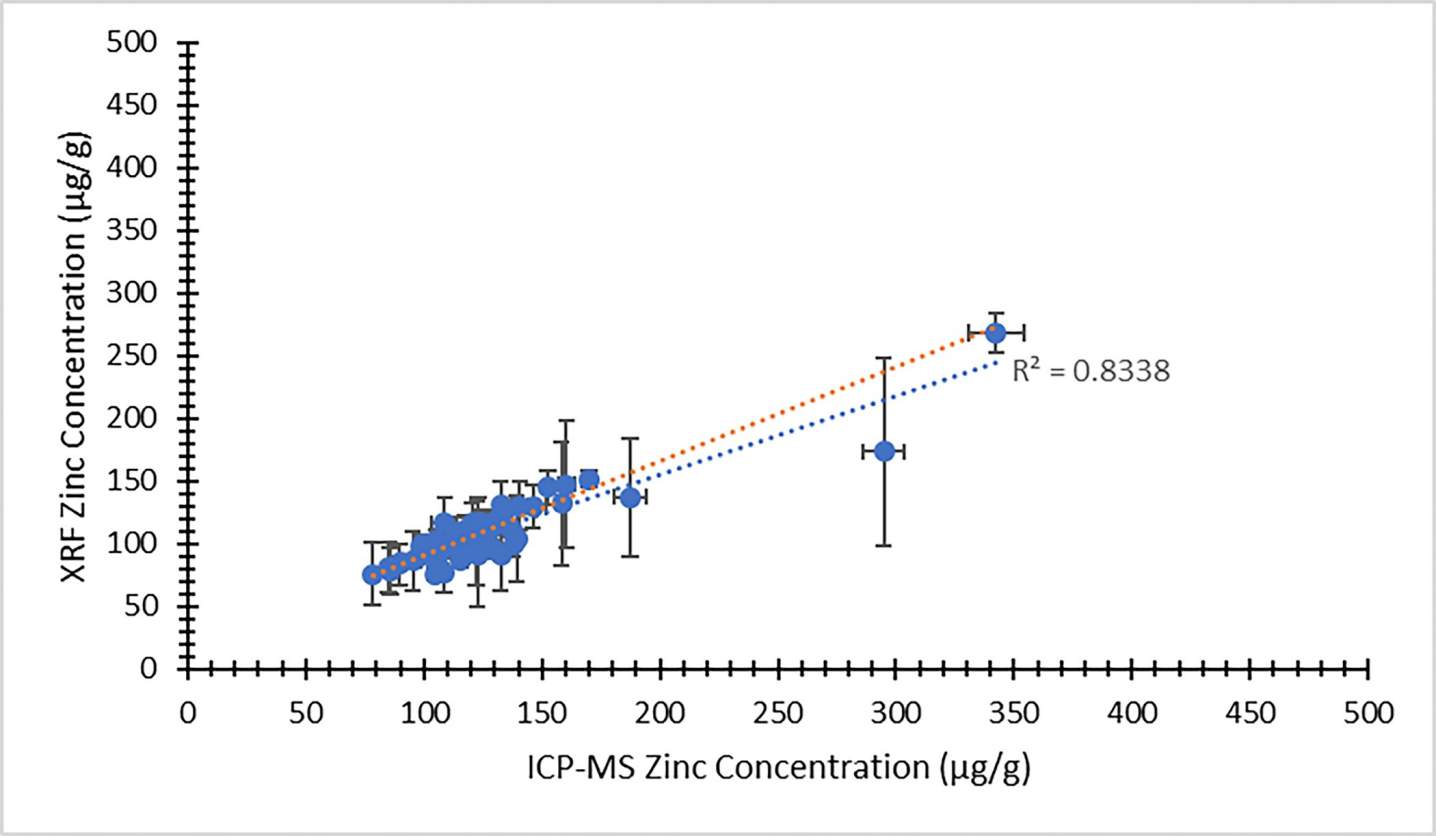
XRF system output zinc concentration (μg/g) as a function of ICP-MS zinc concentration (μg/g) for nail clippings from visit 2 of the MINI study. Uncertainty bars are provided for each data point. The unweighted best fit linear relationship between the variables is displayed as a blue dotted line and the weighted best fit linear relationship is displayed as an orange dotted line.

**Fig 5:**
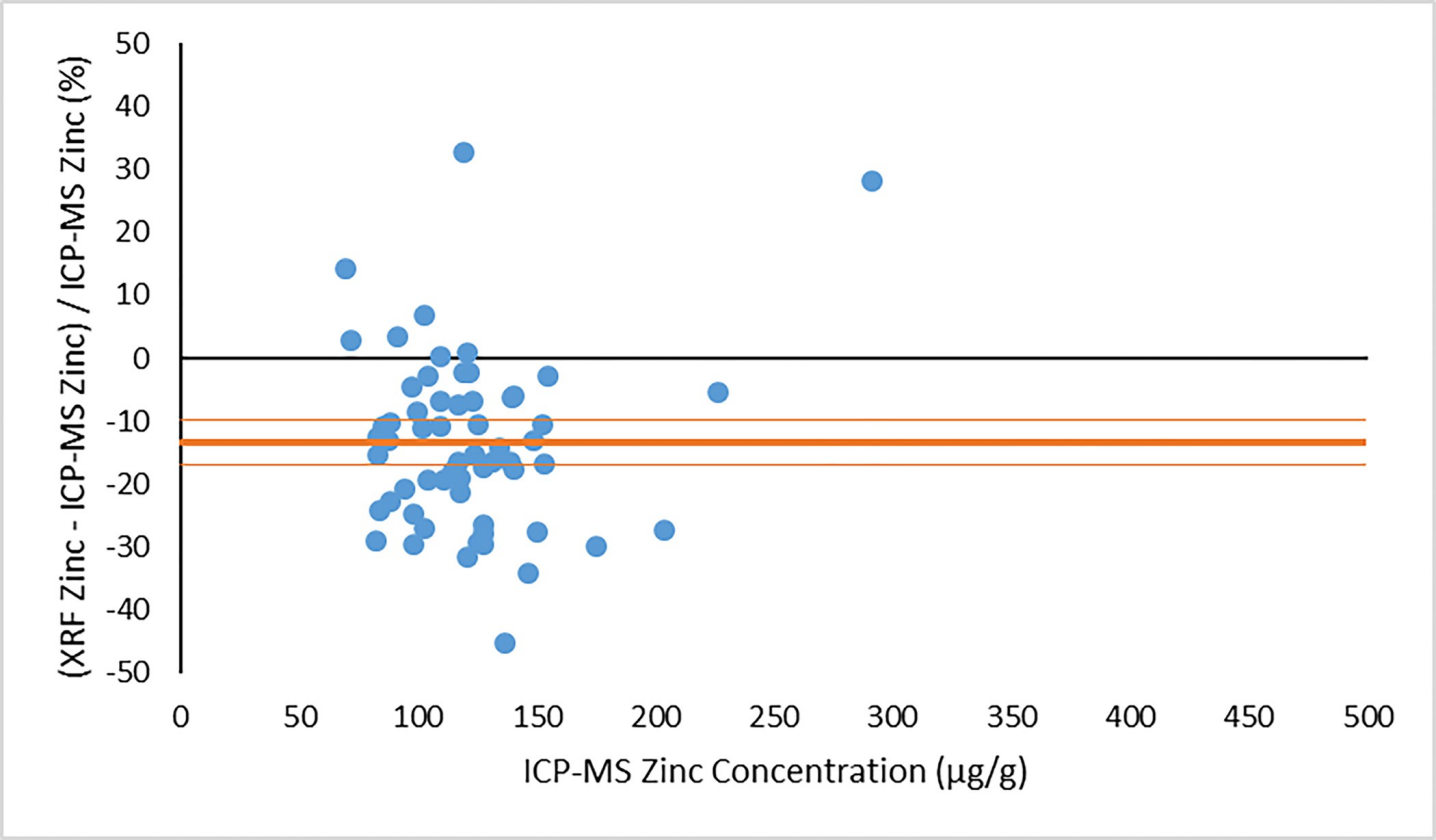
Bland-Altman plot of the percentage difference between XRF and ICP-MS zinc concentration results as a function of ICP-MS zinc concentration from visit 1 of the MINI study. The mean result is given as a bold horizontal line, with confidence interval limits given as lines above and below the mean.

**Fig 6:**
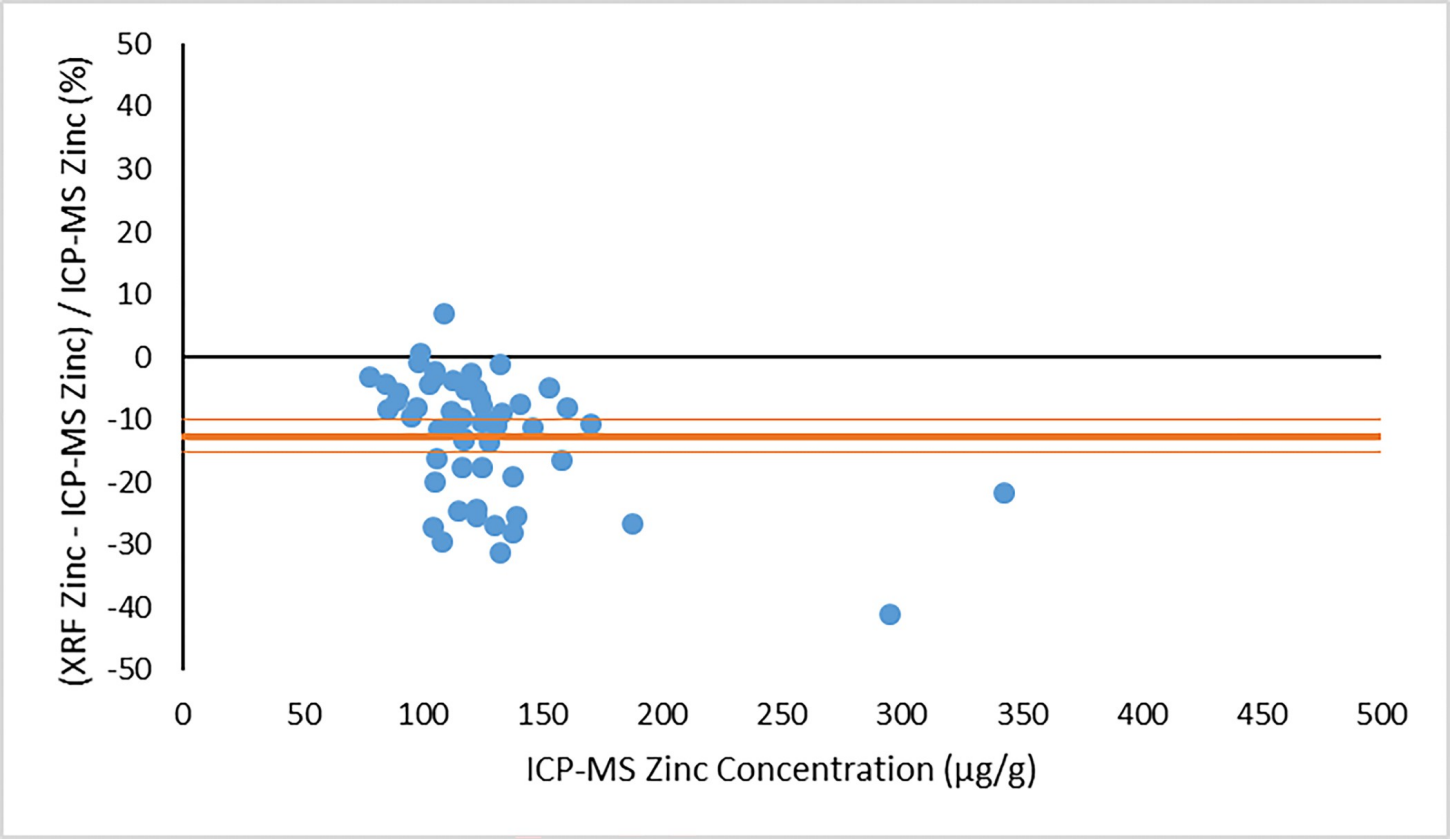
Bland-Altman plot of the percentage difference between XRF and ICP-MS zinc concentration results as a function of ICP-MS zinc concentration from visit 2 of the MINI study. The mean result is given as a bold horizontal line, with confidence interval limits given as lines above and below the mean.

In making a comparison between the XRF results and the ICP-MS concentrations, we consider first the concentration results output directly from the XRF system, using the default factory-calibration. As noted above, these XRF output concentrations are not expected to be accurate (due to matrix effects) but should still scale with the true concentrations [24]. Indeed, for the zinc measurements, the XRF output concentrations correlate quite well with the ICP-MS concentrations, as demonstrated by Figs 3 and 4. Fig 3 illustrates the comparison between XRF system output zinc concentration and ICP-MS zinc concentration from the visit 1 participants. Fig 4 provides the same comparison for the visit 2 data. In both plots, the dependent (y) variable is the weighted average XRF zinc concentration result returned from the four measured nail fragments. The independent (x) variable is the average zinc concentration returned from the ICP-MS analysis. Using the same zinc concentration data obtained from the two methods, a Bland-Altman plot is shown in Fig 5 (visit 1) and Fig 6 (visit 2). For selenium, a large proportion of the XRF concentration results (17% from visit 1 and 36% from visit 2) were reported as having a measurement value below the system detection limit. We therefore do not provide a graphical comparison between XRF selenium concentration results and the ICP-MS selenium concentrations. However, using the energy spectrum analysis software noted above, selenium XRF results were still available from all nail fragments by implementing the TAR analysis method. The zinc and selenium XRF results from this TAR analysis are presented below.

**Fig 7:**
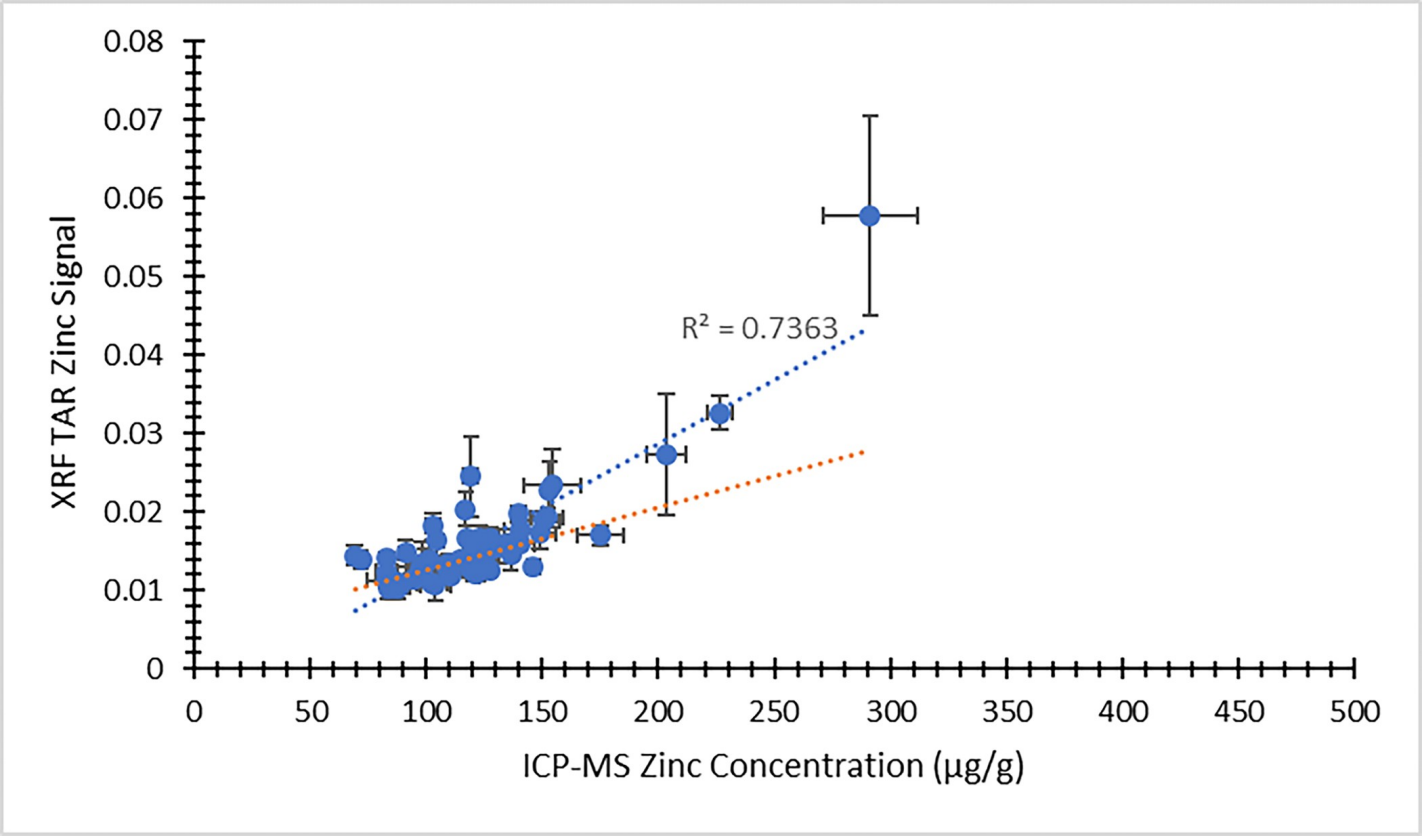
XRF Total Area Ratio (TAR) zinc signal as a function of ICP-MS zinc concentration (μg/g) for nail clippings from visit 1 of the MINI study. Uncertainty bars are provided for each data point. The unweighted best fit linear relationship between the variables is displayed as a blue dotted line and the weighted best fit linear relationship is displayed as an orange dotted line.

**Fig 8:**
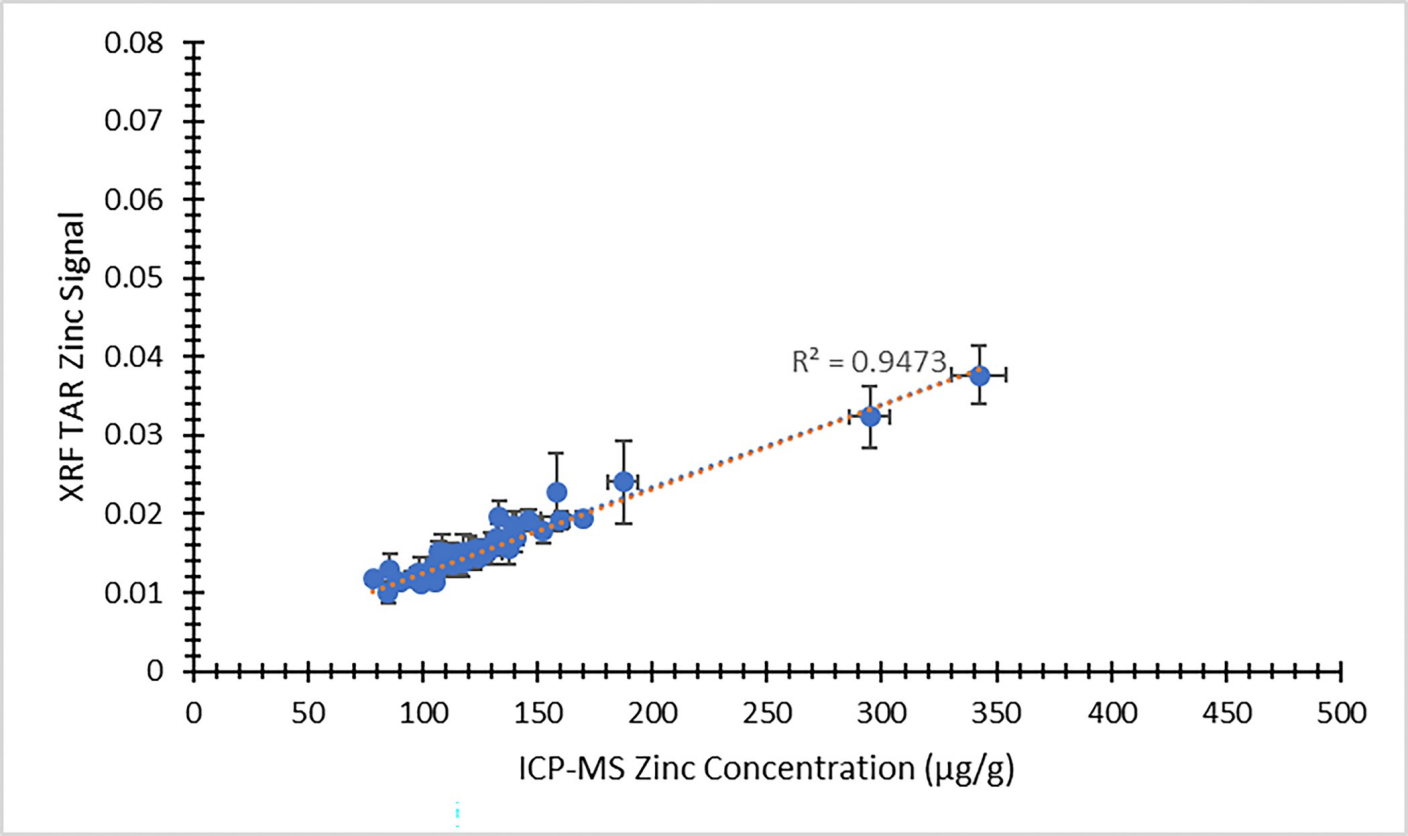
XRF Total Area Ratio (TAR) zinc signal as a function of ICP-MS zinc concentration (μg/g) for nail clippings from visit 2 of the MINI study. Uncertainty bars are provided for each data point. The unweighted best fit linear relationship between the variables is displayed as a blue dotted line and the weighted best fit linear relationship is displayed as an orange dotted line.

**Fig 9:**
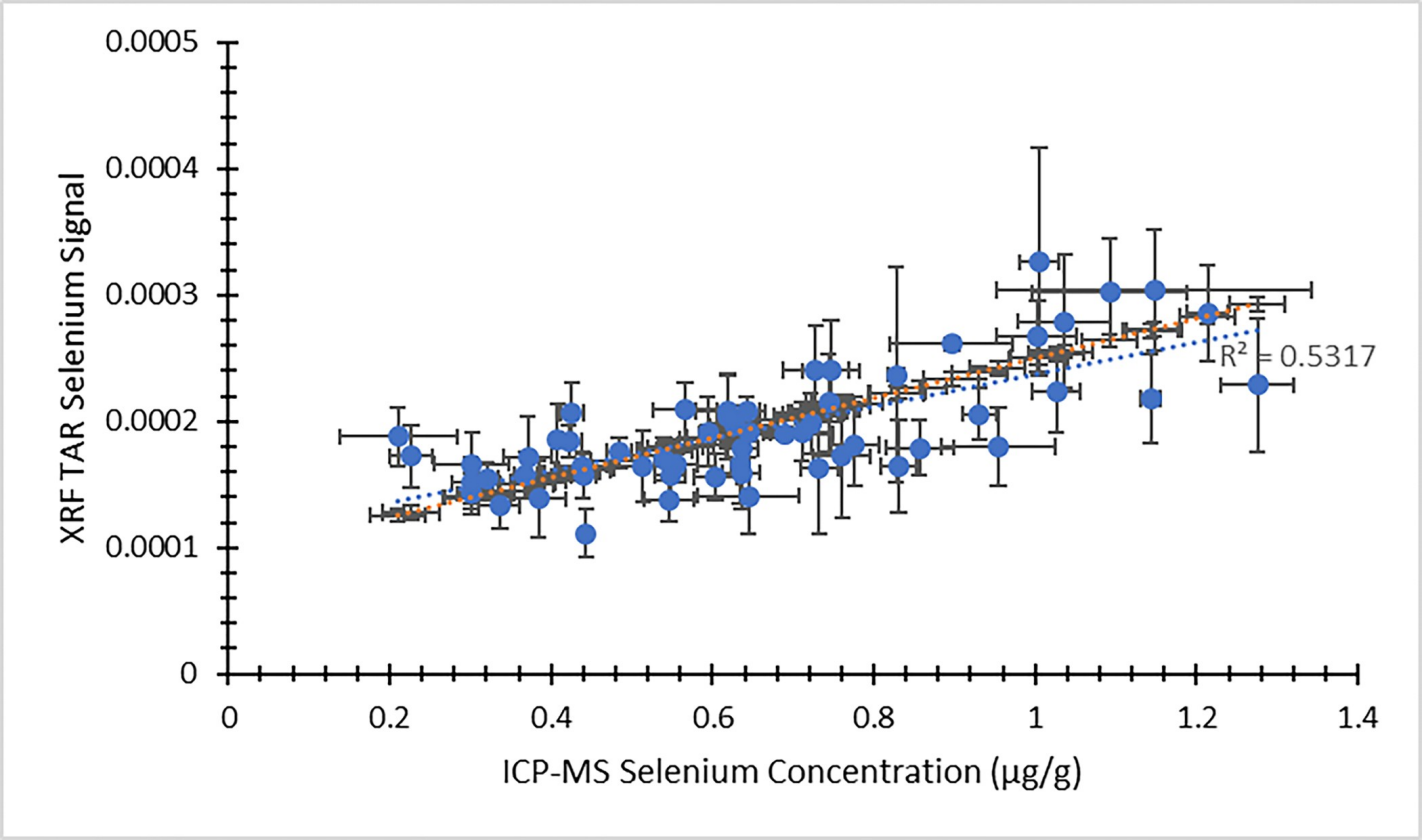
XRF Total Area Ratio (TAR) selenium signal as a function of ICP-MS selenium concentration (μg/g) for nail clippings from visit 1 of the MINI study. Uncertainty bars are provided for each data point. The unweighted best fit linear relationship between the variables is displayed as a blue dotted line and the weighted best fit linear relationship is displayed as an orange dotted line.

**Fig 10:**
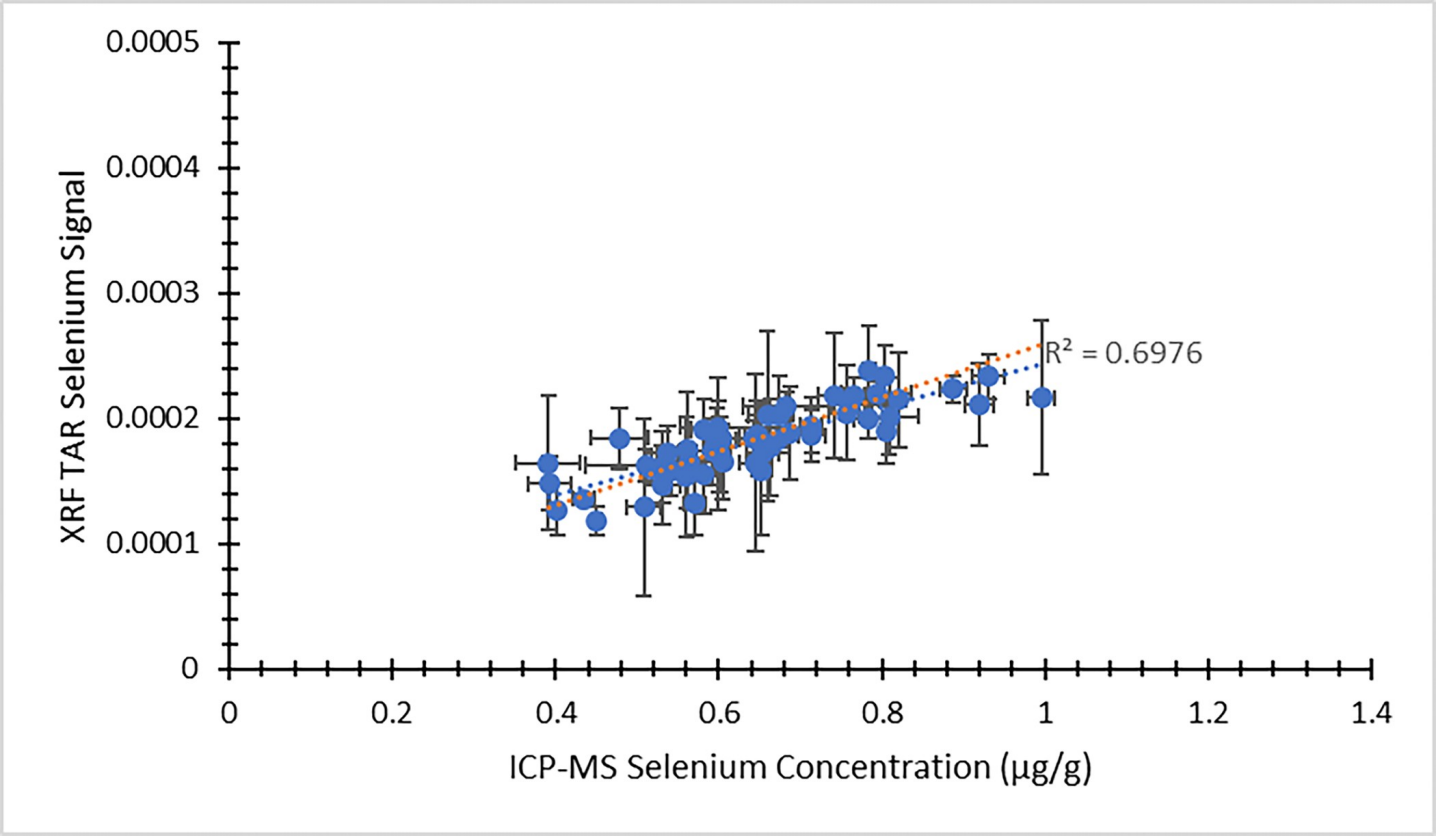
XRF Total Area Ratio (TAR) selenium signal as a function of ICP-MS selenium concentration (μg/g) for nail clippings from visit 2 of the MINI study. Uncertainty bars are provided for each data point. The unweighted best fit linear relationship between the variables is displayed as a blue dotted line and the weighted best fit linear relationship is displayed as an orange dotted line.

Using the XRF energy spectra obtained from individual nail fragments, a second analytical approach was available for comparing the XRF results against ICP-MS concentrations. Fig 7 provides a comparison between the XRF zinc signal using this approach and the ICP-MS zinc concentration from the visit 1 participants. Fig 8 presents the same comparison from visit 2. In both figures, the dependent (y) variable is the weighted average XRF TAR zinc signal from the four nail fragments. The independent (x) variable is the average zinc concentration result returned from ICP-MS. The same approach to XRF spectral analysis was extended to selenium, as well. Fig 9 illustrates the XRF selenium signal as a function of the ICP-MS selenium concentration from the visit 1 participants. Likewise, Fig 10 provides the same comparison from visit 2. Here, the dependent (y) variable is the weighted average XRF TAR selenium signal from the four nail fragments. The independent (x) variable is the average selenium concentration result returned from ICP-MS.

In all cases, the XRF results are expected to scale linearly with the ICP-MS concentration results. Least squares fitting was used to determine equations of best fit for each of the data sets presented in Figs 3–10, and both the unweighted and weighted lines of best fit are indicated in each case. Table 5 provides the slope and y-intercept parameters, along with their uncertainties, for each of the unweighted lines of best fit.

**Table 5:** Linear equation of best fit slopes and y-intercepts for the unweighted data presented in Figs 3–10. Parameter values and their uncertainties are provided in each case.

	Slope	y-intercept
**Fig 3**	1.065 ± 0.075	-23.8 ± 9.6
**Fig 4**	0.629 ± 0.039	29.3 ± 5.2
**Fig 7**	0.000162 ± 0.000013	-0.0037 ± 0.0016
**Fig 8**	0.0001055 ± 0.0000034	0.00232 ± 0.00046
**Fig 9**	0.000127 ± 0.000016	0.000110 ± 0.000011
**Fig 10**	0.000173 ± 0.000016	0.000070 ± 0.000010

## Discussion

The average zinc concentration obtained from ICP-MS measurements of nail clippings in this study ranged from 122 μg/g (visit 1) to 127 μg/g (visit 2), with a median zinc concentration of 118 μg/g from both visits. These values are broadly consistent with nail clipping zinc concentrations observed previously through a variety of international population studies [29–31]. The zinc concentrations seen in the current group of New Zealand mothers and infants were generally higher than those measured recently in a population of adults in Canada, using a similar ICP-MS method [24]. The average selenium concentration observed in nail clippings in this study ranged from 0.646 μg/g (visit 2) to 0.659 μg/g (visit 1). Median selenium concentrations were very similar, with values of 0.635 μg/g for visit 1 and 0.643 μg/g for visit 2. These results fall toward the lower end of nail clipping selenium concentrations observed internationally. For example, Rodushkin and Axelsson [29] noted a mean selenium concentration of 0.830 μg/g from northern Sweden. More recently, a value of 0.364 μg/g was reported from a village in China having low selenium intake, which contrasted with a result of 2.232 μg/g from a village with much higher intake levels [32]. On the other hand, a mean selenium toenail concentration of only 0.52 μg/g was reported for a control population from Northern Ireland and Ireland, measured using INAA [33]. Overall, the relatively low concentration of selenium in nail clippings from the current study may be reflective of the suboptimal selenium intake observed for mothers and infants participating in the MINI study [21].

Uncertainty of measurement is an important consideration for any physical measurement technique. In this study, the relative standard deviation of repeated assessments was used as an indication of measurement uncertainty. Overall, the relative standard deviation of the measurement techniques tended to be smaller for zinc when compared to selenium. This was a natural consequence of the much larger zinc concentrations in the nail clippings. As expected, the gold standard ICP-MS technique had a low relative standard deviation for both zinc and selenium. Notably, however, there appeared to be a consistent difference in these metrics between the visit 1 and visit 2 ICP-MS measurements, with the relative standard deviation being larger for visit 1. This was evident from both the zinc results (Table 3) and the selenium results (Table 4). This observation will be discussed in more detail below. For both analytical approaches used for XRF assessment of the nail clippings (the factory-calibrated output concentrations and the TAR results from energy spectrum analysis), the relative standard deviation was found to be larger for visit 2 than visit 1. This was an expected result since the XRF time of measurement was decreased from 300 s for the visit 1 nail fragments to 180 s for the visit 2 nail fragments. Conversely, as noted, the ICP-MS relative standard deviation was found to be larger for visit 1 than visit 2. The reason for this result is unknown, but the effect was consistent between both the zinc and selenium measurements.

The correlation of the various XRF measurement results with the gold standard ICP-MS concentrations was considered for both zinc and selenium. When using the automated XRF system output zinc concentration values, the correlation with ICP-MS concentration results proved to be strong for both the visit 1 (r^2^ = 0.77) and visit 2 (r^2^ = 0.83) measurements. The Bland-Altman plots, however, highlighted that the automated XRF output for zinc concentration tended to return values that were too low. This reinforced the benefit of performing our own energy spectrum analysis and establishing a line of best fit between the calculated TAR values and the ICP-MS zinc concentrations. When using the XRF energy spectrum analysis TAR approach, the correlation with ICP-MS concentration results remained strong for both visit 1 (r^2^ = 0.74) and, especially, visit 2 (r^2^ = 0.95). It is evident that the placement of individual data points is quite similar between Figs 3 and 7, as well as between Figs 4 and 8. This suggests that, not unexpectedly, the internal routine used to produce the automated XRF system concentrations relies on an analysis approach similar to our own external spectrum analysis. Overall, it was notable that the visit 2 results did not appear to be adversely affected by the decrease in XRF measurement time. This indicates that a 180 s measurement time for each individual nail fragment is a viable approach when using portable XRF for zinc detection. It should be noted, however, that the visit 2 (180 s) measurement comparison benefitted from an especially low relative standard deviation in the ICP-MS results (Table 3). This would serve to improve the correlation between the two measurements. A similar trend was observed from the selenium results. Using the energy spectrum analysis approach, the correlation of XRF results with ICP-MS concentrations showed r^2^ = 0.53 from the visit 1 measurements and r^2^ = 0.70 from the visit 2 measurements. Again, the visit 2 comparison benefitted from an especially low relative standard deviation in the ICP-MS results (Table 4). While the comparison between XRF and ICP-MS certainly revealed greater scatter when assessing selenium (relative to zinc), the selenium results were surprisingly strong given the relatively low concentrations of selenium in nail clippings. For both zinc and selenium, equations of best fit established in this study from the plotting of TAR results against ICP-MS concentrations could be used as calibration lines for future XRF nail clipping measurements. In this way, XRF analysis of new nail clippings with the TAR approach could be used to determine concentrations through the application of an appropriate calibration line.

It is evident from Table 5 and the corresponding figures that the equations of best fit tended to return significant non-zero (and usually positive) y-intercept results from both the zinc and selenium comparisons. This effect was slightly more prominent for the selenium results relative to the zinc results. Observation of a similar y-intercept term was made in a previous study [24], with a variety of potential explanations noted. The existence of a contaminating non-zero background signal at zero concentration was ruled out in the case of zinc [24], and this does not appear to be the cause. Viable explanations could include effects from inhomogeneous distributions of elements within nail clippings, and/or effects from different thicknesses (or other physical parameters) between clippings. These considerations require further study. It is worth remarking that the non-zero y-intercept terms in the current study and the previous study [24] were observed from both the automated XRF concentration results and the XRF energy spectrum TAR results. The effect is therefore not unique to the TAR approach.

For the zinc levels assessed from nail clippings in the current study, both approaches to XRF analysis provided stronger correlations with ICP-MS zinc concentration than were observed in a recent study using similar methods [24]. This previous study exhibited a coefficient of determination of r^2^ = 0.37 when using the automated XRF system output zinc concentrations, and r^2^ = 0.46 when using the XRF energy spectrum TAR approach. The explanation for the general improvement in the current study is likely multi-faceted. The previous study made use of three individual 300 s XRF measurements, all performed on the same spot of the same nail clipping. By moving to spot measurements on four different clippings, the current XRF study protocol may have sampled a nail volume which is more representative of the whole and less susceptible to bias from small-scale variations in zinc concentration [23]. As well, the previous study performed ICP-MS analysis from only a single clipping having, on average, a low mass of 3.7 mg [24]. By sampling additional clippings, the ICP-MS measurements in the current study may have benefited from a slightly improved accuracy and precision. The improved correlation between XRF zinc TAR results and ICP-MS zinc concentrations observed from visit 2 of this study was roughly similar to that observed using a different portable XRF technique (r^2^ = 0.99) with a collection of toenail clippings spanning a broader range of concentrations [34].

While selenium has not been analyzed previously using the portable XRF method outlined here, previous results involving arsenic are available [25]. Since selenium and arsenic have similar atomic numbers, characteristic X-ray energies, and concentrations in human nail, a comparison between results from the two elements is relevant. When examining XRF energy spectrum TAR arsenic results as a function of ICP-MS arsenic concentration, a coefficient of determination of r^2^ = 0.59 was observed [25]. This degree of correlation from the arsenic results is similar to those seen in the current study for selenium results from both the visit 1 and visit 2 data sets. Taken together, these two studies suggest that simultaneous measurement of arsenic and selenium in nail clippings is possible using portable XRF. This could prove of benefit for future studies involving nail as a biomarker, especially since there is recent evidence of an antagonistic relationship between selenium and arsenic in the human body, suggesting a possible protective role for selenium [35]. The correlation between the XRF selenium TAR results and ICP-MS selenium concentrations observed from visit 2 of the current study was slightly stronger than those reported using a different portable XRF technique (r^2^ = 0.55) [34] and a benchtop XRF system (r^2^ = 0.60) [36] with toenail clippings having concentrations less than 2 μg/g.

## Conclusions

This study has investigated the potential for portable XRF to measure zinc and selenium in nail clippings, through an application involving a subset of women and infants participating in the MINI study. The focus of this paper has largely been on methodology and the development of recently introduced portable XRF approaches for nail clipping analysis. A strength of this study is the use of nail clippings from both mothers and their infants, providing a wide range of clipping masses and sizes. The extension of the portable XRF method to the measurement of selenium is another strength. A potential weakness of the study is the relatively limited number of measurements available for comparison between the XRF and ICP-MS results. Future work involving the current data set will consider other factors such as whether any changes in zinc or selenium nail clipping levels were observed between visit 1 and visit 2 for either the mothers or infants, how other indices of intake relate to the nail clipping results, and whether any related health outcomes are evident. Along the same lines of investigation, further measurement results for the same mother-infant pairings will be available from visit 3 (12 months postpartum), allowing a fuller picture of time development in this population.

In summary, the current study demonstrated improved capabilities for the assessment of zinc in nail clippings using portable XRF. As well, for the first time, the same portable XRF technique was successfully applied for the measurement of selenium in nail clippings. When comparing XRF results with ICP-MS concentration results, it appears that an XRF energy spectrum analysis approach provides slightly superior results over using the XRF system output concentrations taken directly from the unit. The energy spectrum approach does require additional user expertise and analysis time, and for future applications would necessitate the use of an established equation of best fit for a particular element to convert a result obtained from the spectrum to a concentration. For the measurement of zinc concentration, especially, the portable XRF technique appears capable of providing accurate results with a reliable level of precision. The XRF method outlined here may have potential for future applications to trace element detection in nail clippings where factors such as measurement portability, speed, or cost are especially important.

## Supporting information

S1 Data (XLSX)

S2 Data (XLSX)
